# Successful use of ultraslow thrombolytic therapy in stuck mechanical aortic valve in a patient with COVID-19; a case report

**DOI:** 10.1016/j.ijscr.2022.107233

**Published:** 2022-05-20

**Authors:** Sumaya Al Helali, Hassan Sandokji, Abdurahamn Al Moughari, Hamid Al Ghamdi, Turki Assiri, Hussain Al Amri

**Affiliations:** Prince Sultan Cardiac Centre, Riyadh, Saudi Arabia

**Keywords:** Ultraslow thrombolytic therapy, Stuck mechanical aortic valve, COVID-19, Saudi Arabia

## Abstract

**Introduction and importance:**

COVID-19 represents a new challenge for patients with prosthetic valve, through increasing the risk of thrombosis and reducing the frequency of anticoagulation follow up visits.

**Case presentation:**

A 37-year-old male patient on aspirin and warfarin for a mechanical aortic valve (AV, St Jude size 21 mm), presented with generalized fatigue and loss one of the mechanical heart sounds for 10 days. Urgent fluoroscopy showed stuck one of the AV leaflets in a closed and opening positions. Echocardiography showed high peak and mean AV gradients. The patient was confirmed with COVID-19 with fever on the day of admission. Cardiac CT with contrast showed stuck right (posterior) disc with a 6 × 4 mm thrombus surrounded by pannus formation. The patient was started on ultraslow thrombolytic therapy (alteplase 1 mg, every hour for 25 h, followed by 6 h of unfractionated heparin). Repeated fluoroscopy showed normal opening and closure of both discs. Repeated echocardiography showed a significant reduction in the peak and mean AV gradients back to baselines. The patient was discharged after 7 days with INR 3.0 for two consecutive days. The patient was asymptomatic with stable INR in three- and six-month follow-up visits. Transthoracic ultrasound demonstrated normally functioning mechanical AV.

**Clinical discussion:**

Accurate and timely diagnosis of stuck mechanical AV requires high suspicion and timely diagnostic aids.

**Conclusion:**

Full recovery can be achieved after one cycle of ultraslow thrombolytic therapy. Further supportive data are still needed before recommending thrombolytic therapy as a successful alternative to surgery in COVID-19 patients.

## Highlights

• Few case reports linked COVID-19 to thrombosis of mitral/aortic valve prostheses.
• Stuck aortic valve prosthesis in a patient with COVID-19 can be accidently diagnosed.
• Accurate and timely diagnosis requires high suspicion and timely diagnostic aids.
• Full recovery can be achieved after one cycle of ultraslow thrombolytic therapy.
• Still too early to recommend thrombolytic therapy over surgery in COVID-19 patients

## 1 Introduction

COVID 19 infection considerably increases the risk of thrombotic complications, mainly venous and to less extent arterial ones [1], [2]. The prevalence of venous thromboembolic events was estimated at 13% to 15% among hospitalized patients and up to 30% among ICU patients with severe disease [1], [2]. The prevalence of arterial thromboembolic events was estimated at 4% [1]. The increase in COVID-19 related thrombosis largely happens despite prophylactic anticoagulation therapy [3]. The underlying pathophysiology is not fully understood. However, it is believed that the coagulopathy is triggered by the COVID-19 related hyperinflammatory response known as “cytokine storm” and endothelial damage caused by several direct and indirect factors [4], [5]. The coagulopathy is immune-mediated and involves activations of complement, platelets, extracellular neutrophil traps, and coagulation system [4], [5].

COVID-19 represents a new challenge for patients with prosthetic valve. COVID-19 related restriction to routine coagulation visits for non-COVID-19 patients may result in increase in the rates of stuck valves [6]. Additionally, few case reports of COVID-19 associated thrombosis of aortic valve (AV) prosthesis [7], [8] and mitral valve (MV) prosthesis [9], [10], [11], [12] have been published. The above patients had biological prosthesis and some were complicated with thromboembolic events at presentation [8], [10]. Here we report a case of stuck mechanical AV in a patient with COVID-19 that was successfully treated by ultraslow thrombolytic therapy. The case is reported in compliance with the surgical case report (SCARE) guidelines for 2020 [13].

## 2 Case report

**Fig. 1:**
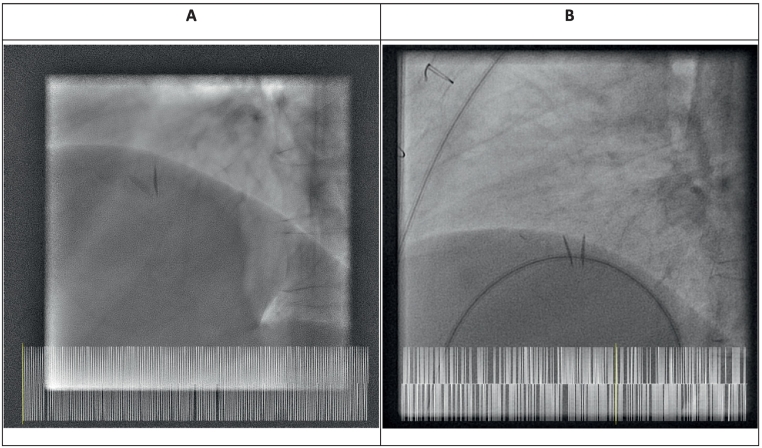
Fluoroscopy showed stuck one of the AV leaflets at baseline (A) which was normally functioning one days after starting the thrombolytic therapy (B).

**Fig. 2:**
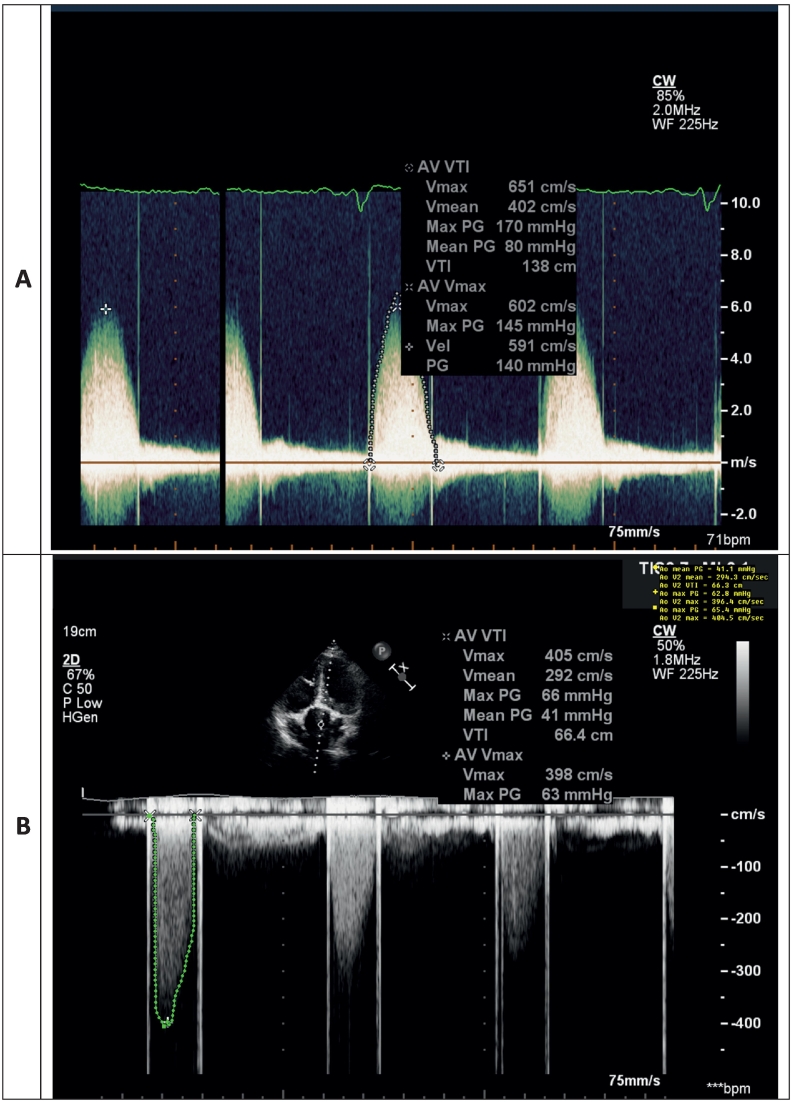
Echocardiography showed high peak and mean AV gradients (170 and 80 mm Hg, respectively) on the first day of admission (A) and reduced peak and mean AV gradients (66 and 41 mm Hg, respectively) on the 2nd day of admission (B).

**Fig. 3:**
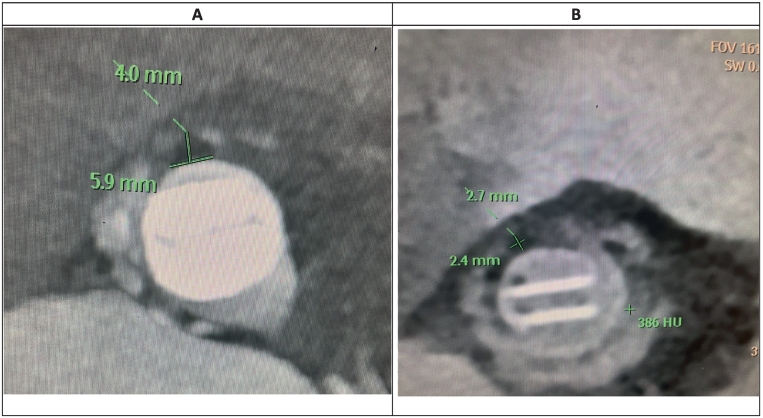
CT with contrast at baseline showed stuck right (posterior) disc with a thrombus (6 × 4 mm) surrounded by pannus formation (A) while repeat study after one cycle of thrombolytic therapy there was reduction in the thrombus size (2.7 × 2.4 mm) (B).

**Fig. 4:**
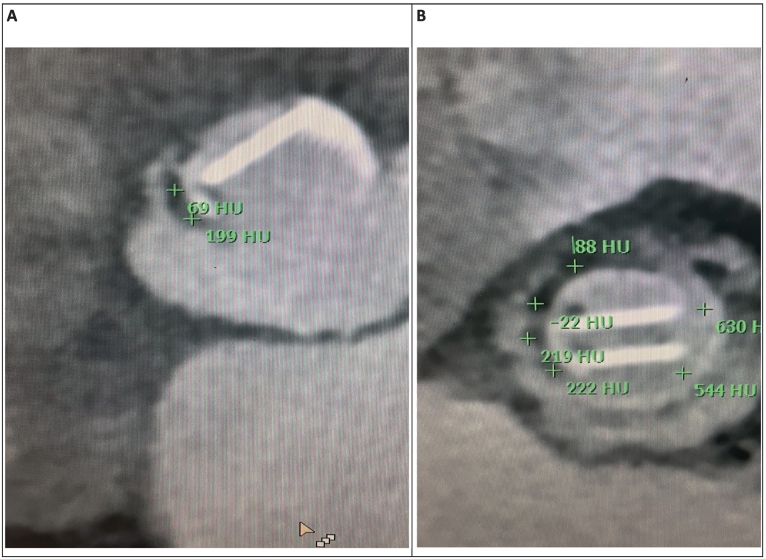
CT with contrast showed the attenuation of thrombus < 90HU surrounded by pannus formation with CT attenuation >145 HU: pre thrombolytic therapy (A) and post the single cycle of lytic therapy (B).

A 37-year-old male patient with mechanical AV (St Jude size 21 mm) was presented to the cardiology clinic during a regular follow up visit with generalized fatigue and loss of one of the mechanical heart sounds for 10 days duration, with no history of stopped warfarin or aspirin but has less frequency of anticoagulation visits. The patient had the mechanical AV inserted in 1998 after diagnosis of severe rheumatic aortic regurgitation, with baseline transvalvular mean gradient of 40 mm Hg. Physical examination showed normal level of consciousness, normal vital signs, and sinus rhythm. Cardiologic examination confirmed the absence of second heart sound. Chest and abdomen were unremarkable. Lower limbs showed no edema nor signs of deep venous thrombosis (DVT). The patient was admitted and had a routine nasopharyngeal swab for COVID-19 test. The patient then underwent urgent fluoroscopy which showed stuck one of the AV leaflets in a closed and opening positions (Fig. 1A). Same day echocardiography showed well-seated AV with high peak and mean AV gradients (170 and 80 mm Hg, respectively) and moderate intrinsic aortic regurgitation (AR), (Fig. 2A). Additionally, there were moderate mitral and tricuspid regurgitation. Ejection Fraction was 50% and pulmonary artery systolic pressure was 50 mm Hg. The management plan was to redo AV replacement (AVR) and the patient was prepared for transesophageal echocardiography (TEE). However, the COVID-19 test came positive on the second day of admission with documented fever. Therefore, the patient was transferred to a specialized COVID-19 unit, surgical plan was replaced by ultraslow thrombolytic therapy, and the TEE was replaced with multidetector–row computed tomography (MDCT) with contrast. The later showed stuck right posterior disc with a thrombus size 5.9 mm × 4 mm (area 15.5 mm^2^) at the hinge point of fixed disc into the supra-valvular area, it has CT attenuation of 60 HU surrounded by pannus formation which is partially enhanced and CT attenuation of 194 HU (Figs. 3A & 4A). Laboratory findings showed platelets count of 317 1000/μL, prothrombin time (PT) of 15 s, activated partial thromboplastin time (aPTT) of 36 s, international normalized ratio (INR) of 1.1, C-reactive protein (CRP) of 7.0 mg/L, ferritin of 646 ng/mL, and D-dimer of 372 ng/mL. The treating cardiologist started the patient on ultraslow thrombolytic therapy following the regimen of PROMETEE trial [14]; alteplase 1 mg, every hour for 25 h, followed by 6 h of unfractionated heparin (70 u/kg bolus then 16 u/kg/h with a target aPTT of 1.5 to 2.0 times the control value). Repeated fluoroscopy showed normal opening and closure of both discs (Fig. 1B). Repeated echocardiography showed a significant reduction in the peak and mean AV gradient back to their baselines (66 and 41 mm Hg, respectively, Fig. 2B). Repeated cardiac CT done 48 h after starting the thrombolytic therapy showed a reduction of the thrombus size to 2.7 × 2.4 mm (Figs. 3B & 4B). The patient was discharged after 7 days with INR 3.0 for two consecutive days and continued aspirin. Before discharge, the patient was strongly advised to regularly follow up with the anticoagulation clinic. The patient had two follow up visits at three- and six-month post-discharge. The patient was asymptomatic with stable INR in both visits. Additionally, transthoracic ultrasound done at the three-month follow-up visit demonstrated normally functioning mechanical AV.

## 3 Discussion

The Prosthetic valve thrombosis is extremely rare complication of AVR, with an annual incidence of 3 to 13 per 10,000 patients [15]. Diagnosis and management of stuck mechanical AV in patients with COVID-19 is not well characterized. A number of recent case reports described COVID-19 related stuck MV prosthesis [9], [10], [11], [12]. Additionally, a couple of reports involving bioprosthetic AV thrombosis in COVID-19 patients have been reported outside Saudi Arabia [7], [8]. Unlike the current patient who had long standing mechanical AV, previously reported AV thrombosis was described in old patients who had a recent AVR using biological prosthesis [7], [8].

The diagnosis of the current patient was largely accidental due to lack of associated thromboembolic complications. The diagnosis of the current patient was in the middle of the second wave of COVID-19 in Saudi Arabia (April to August 2021) [16]. Although the access to cardiac healthcare services was not restricted during the second wave as compared with the first wave, cardiac patients are frequently reluctant to attend their scheduled follow up visits over concerns of infection [6]. The current report highlights inadequate follow up of anticoagulation therapy in cardiac patients with prosthetic valves during the COVID-19 era [6]. This COVID-19 related challenge may require non-traditional strategies such as frequent patient reminders and use of telemedicine clinics. Additionally, accurate and timely diagnosis requires the cardiologist to have high rate of suspicion and timely diagnostic aids [11].

The current patient was successfully treated using one cycle of ultraslow thrombolytic therapy. The same regimen was successful in managing 90% of patient with stuck mechanical valves, especially among those without atrial fibrillation, low NYHA class, and small thrombus area [14]. Recently, alteplase was successfully used in managing COVID-19-related MV thrombosis [12]. Recent guidelines of the American College of Cardiology/American Heart Association (ACC/AHA) recommend either slow-infusion low-dose thrombolytic therapy or emergency surgery as first-line treatment strategies for the management of patients with left-sided mechanical prosthetic valve thrombosis [17]. The current finding is probably suggestive of the high anti-inflammatory effect of alteplase in patients with COVID-19 related immunothrombosis. TEE was not used in evaluating the patient because other non-aerosol generating diagnostic modalities was sufficient to clearly evaluate the diagnosis and prognosis [18]. Meta-analysis suggested that MDCT and 3D TEE have higher sensitivity than do TTE and 2D TEE, and can be reliable imaging modalities for detecting a sub-prosthetic mass that causes prosthetic valve obstruction (PVO) [17]. Moreover, MDCT can more accurately differentiate the cause of PVO than does TEE [6]. Interestingly, anticoagulation therapy in the form of heparin and warfarin infusion was successful in managing AV thrombosis in the two patients with COVID-19 mentioned above [7], [8]. Although promising, further supportive data are still needed before recommending thrombolytic therapy as a successful alternative to surgery in COVID-19 patients.

## Consent

Written informed consent was obtained from the patient for publication of this case report and accompanying images. A copy of the written consent is available for review by the Editor-in-Chief of this journal on request.

## Provenance and peer review

Not commissioned, externally peer-reviewed.

## Ethical approval

Case reports are exempted from ethnical approval as per the guidelines of the research and ethics committee at the Prince Sultan Cardiac Center in Riyadh, Saudi Arabia.

## Funding

All authors received no financial support related to this research.

## Guarantor

Sumaya Al Helali, MD.

## Research registration number

Not applicable.

## CRediT authorship contribution statement

**Sumaya Al Helali**, cardiologist treating the patient, idea, first draft, and submission; **Hassan Sandokji**, help in writing, references, and critical review of final version; **Abdurahamn Al Moughari**, help in writing, references, and critical review of final version; **Hamid Al Ghamdi**, help in writing, references, and critical review of final version; **Turki Assiri**, help in writing, references, and critical review of final version; **Hussain Al Amri**, help in writing, references, and critical review of final version.

## Declaration of competing interest

All authors have nothing to declare.
